# *In silico* Analyses of Subtype Specific HIV-1 Tat-TAR RNA Interaction Reveals the Structural Determinants for Viral Activity

**DOI:** 10.3389/fmicb.2017.01467

**Published:** 2017-08-08

**Authors:** Larance Ronsard, Tripti Rai, Devesh Rai, Vishnampettai G. Ramachandran, Akhil C. Banerjea

**Affiliations:** ^1^Laboratory of Virology, National Institute of Immunology New Delhi, India; ^2^Department of Microbiology, University College of Medical Sciences and Guru Teg Bahadur Hospital New Delhi, India; ^3^Department of Gastroenterology and Human Nutrition, All India Institute of Medical Sciences New Delhi, India; ^4^Department of Microbiology, All India Institute of Medical Sciences New Delhi, India

**Keywords:** HIV-1 Tat, transactivation, TAR RNA, genetic variations, molecular docking, hydrogen bond interaction

## Abstract

HIV-1 Tat transactivates viral genes through strong interaction with TAR RNA. The stem-loop bulged region of TAR consisting of three nucleotides at the position 23–25 and the loop region consisting of six nucleotides at the position 30–35 are essential for viral transactivation. The arginine motif of Tat (five arginine residues on subtype TatC) is critically important for TAR interaction. Any mutations in this motif could lead to reduce transactivation ability and pathogenesis. Here, we identified structurally important residues (arginine and lysine residues) of Tat in this motif could bind to TAR via hydrogen bond interactions which is critical for transactivation. Natural mutant Ser46Phe in the core motif could likely led to conformational change resulting in more hydrogen bond interactions than the wild type Tat making it highly potent transactivator. Importantly, we report the possible probabilities of number of hydrogen bond interactions in the wild type Tat and the mutants with TAR complexes. This study revealed the differential transactivation of subtype B and C Tat could likely be due to the varying number of hydrogen bonds with TAR. Our data support that the N-terminal and the C-terminal domains of Tat is involved in the TAR interactions through hydrogen bonds which is important for transactivation. This study highlights the evolving pattern of structurally important determinants of Tat in the arginine motif for viral transactivation.

## Introduction

Human immunodeficiency virus type 1 (HIV-1) generates enormous mutations leading to generation of highly virulent variants and recombinants in an infected population (Ho et al., 1995; Buonaguro et al., 2007). These variants could affect both the host and the viral functional activities (Blackard et al., 2002) resulting in high pathogenesis to AIDS despite antiretroviral therapy (ART) (Geretti, 2006; Kirchhoff, 2009; Sharp and Hahn, 2011; Santoro and Perno, 2013). The trans-activator of transcription (Tat) is involved in the viral transcription from long terminal repeat (LTR) promoter via interaction with trans-activation response element (TAR) sequence at the 5' end of the LTR (+1 to +59) (Dingwall et al., 1989; Buonaguro et al., 1994). Tat binds to TAR and host factors like Cdk9 and cyclin T1, and then recruits various transcriptional factors including a positive transcription elongation complex (P-TEFb), an elongation factor composed of cyclin T1 (CycT1) and Cdk9, which in turn phosphorylates RNA polymerase II resulting in an increased transcription of viral genes (Zhou and Rana, 2002).

TAR interaction with Tat is critical for efficient transcription of viral genes (Rana and Jeang, 1999; Karn and Stoltzfus, 2012). TAR forms a stable stem-loop structure in which a key element is a 3-nucleotide bulge (UCU; position 23–25) (Roy et al., 1990; Davidson et al., 2009). Tat binds directly to this bulged region (Berkhout et al., 1989; Selby et al., 1989). The loop region in TAR (position 30–35) is also required for transactivation (Aboul-ela et al., 1996; Wemmer, 1996). In the case of Tat, the N-terminal region is not directly involved in TAR interaction, however, it is required for viral transactivation (Demarchi et al., 1999). Motifs present in Tat involved in this interaction includes a short (9 residues) sequence of basic amino acids, in which arginine (Arg) residues mediate specific recognition of TAR (Demarchi et al., 1999). In particular, Arg52, Agr56, and Arg57 are critically important for transactivation (Edwards et al., 2005), while any changes in these mutations namely Arg52Gly, Arg56Gln, Arg57Gly, Ser62Gly, and Thr64Asp could lead to reduced TAR interaction and decreased viral transactivation (Pantano et al., 2002; Turk et al., 2006).

The subtype C is predominant in India with the emergence of various recombinants in various HIV-1 genes (Neogi et al., 2011; Ronsard et al., 2015). Due to rapid evolution of HIV-1 strains in India, it is essential to understand the role of structural determinants of HIV-1 Tat in relation to TAR interaction. Our previous data revealed that the genetic variations in Tat could lead to differential levels of LTR-mediated transcription (Ronsard et al., 2014) through strong interaction with TAR RNA *in vitro* (Ronsard et al., 2017); however, the structurally important residues of Tat involved in the interaction with TAR have not been well explored both in relation to the wild type Tat and the mutants. Therefore, it is essential to decipher the role of structural determinants of Tat for TAR interaction and also find out the subtype specific differential interactions of Tat with respect to TAR.

Here, we report the structurally important residues of Tat for enhanced LTR transactivation when compared to wild-type Tat. Natural mutant Ser46Phe exhibited more hydrogen bond interactions with TAR than the wild type Tat (that lacked Ser46Phe) by molecular docking. This study illustrates the number of hydrogen bond interactions formed between Tat and TAR. And also, the data from this study reveals that apart from TAR binding domain, the N-terminal and the C- terminal domains of Tat is also involved in TAR interaction. We report the subtype B and C specific differential Tat and TAR interaction via hydrogen bond interactions. This report demonstrates the structurally evolved Tat residues for viral transcription in comparison to the wild type Tat C.

## Results

### Selection of tat variants for molecular docking with TAR

**Figure 1:**
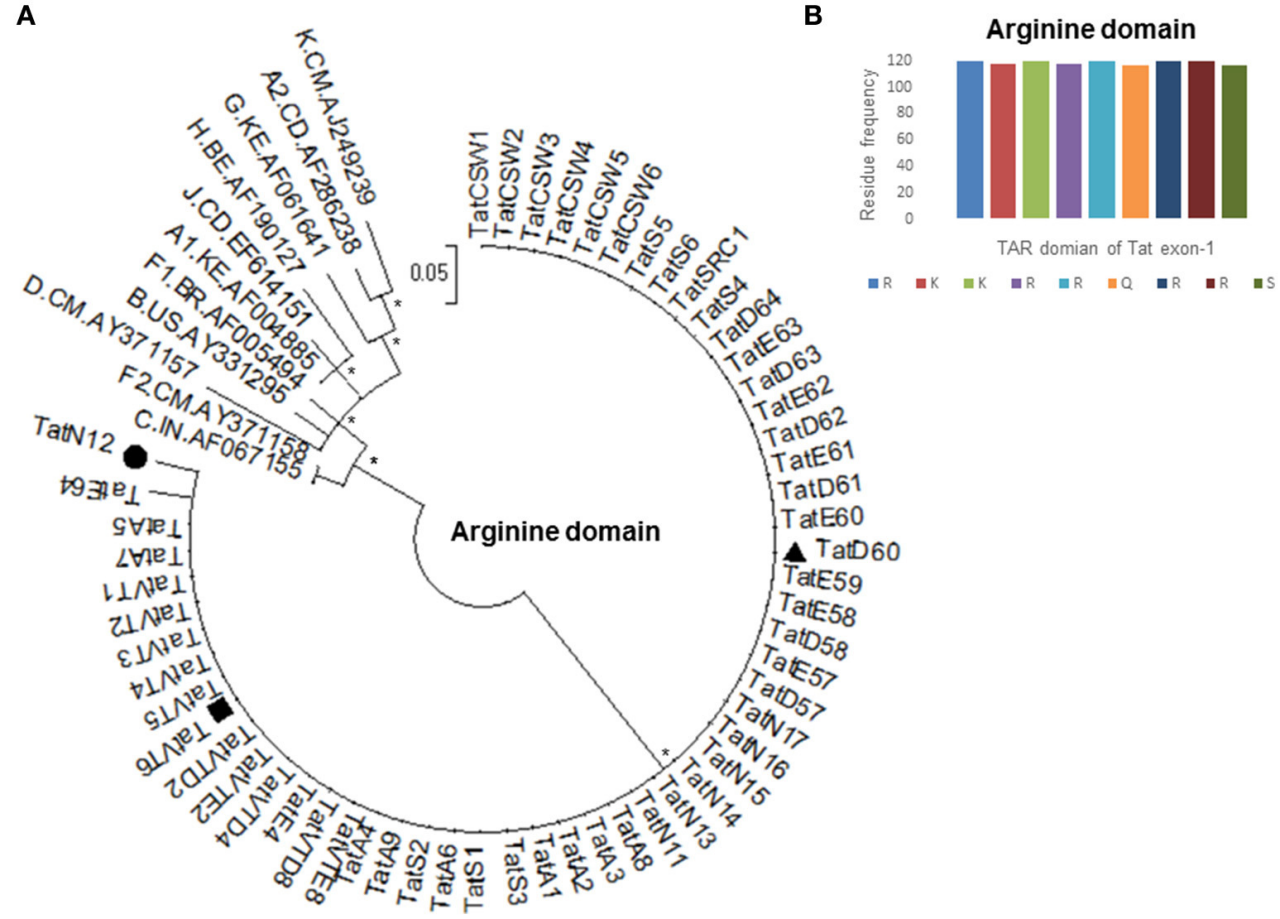
Amino acid conservation of TAR domain of Tat exon-1. **(A)** Phylogenetic tree of Tat variants with M group reference subtypes (A to K including A1, A2, F1, and F2). Each reference sequence was labeled with subtype, followed by the country of isolation and accession number. Filled circle represents TatN12, filled rectangle represents TatVT6 and filled triangle represents TatD60. Mega Version 6 is used for construction of phylogenetic tree with the bootstrap probability (>60%, 1,000 replicates) indicated with an asterisk (^*^) at the corresponding nodes of the tree and the scale bar represents the selection distance of 0.05 nucleotides per position in the sequence. **(B)** Amino acid signature pattern of TAR domain of Tat exon-1 variants was compared with Indian subtype C Tat (Accession number AF067155) sequence. The X-axis represents the amino acid consensus sequence of Tat C with the Arginine rich (49–57 aa) domain and the Y-axis represents the amino acid frequency observed in North Indian Tat variants.

Based on our previous study (Ronsard et al., 2017) on 120 HIV-1 patients, three Tat variants (TatN12, TatD60, and TatVT6) were selected for Tat-TAR docking experiments based on their similarity in mediating LTR transactivation and having similar pattern of mutations in the Tat gene. TatN12 (subtype C) has Leu35Pro and Gly44Ser mutations but lacked Ser46Phe, TatD60 (subtype C) has Glu9Lys, Ser46Phe and Ser61Arg mutations, and TatVT6 is a B/C recombinant but lacked Ser46Phe. The phylogenetic tree was constructed with arginine motif (TAR motif) sequences of Tat variants to show that the TAR motif was highly conserved among North Indian population (Figure 1A). The amino acid sequence pattern analysis (Figure 1B) also showed that the TAR motif was highly conserved with no changes in the arginine and lysine residues indicating the functional importance of these residues in this motif in mediating Tat functions.

### Tat mutations alter interacting ribonucleotides of TAR

To probe whether Tat mutations affect the binding efficiency of Tat toward TAR, *in silico* molecular docking was performed for Tat variants with TAR. Tat is known to interact with multiple host factors that ensure binding affinity of Tat to TAR, here we focused only on the major interacting partner TAR to understand how Tat variants result in varying levels of transactivation. Wild-type TatC-TAR was treated as a baseline level of binding to compare with the mutant subtype C Tat variants. We made homology models of Tat variants (Figure S1) using Modeller 9v8 software. Tat variants were then docked with TAR to predict the binding residues using the HADDOCK web server (Dominguez et al., 2003). The region spanning 17–45 nt of TAR (crystal structure PDB ID: 1ANR) was used for docking; this region encompasses the bulge (+23 to +25) known to be the binding site for Tat protein with TAR (Dingwall et al., 1990).

**Figure 2:**
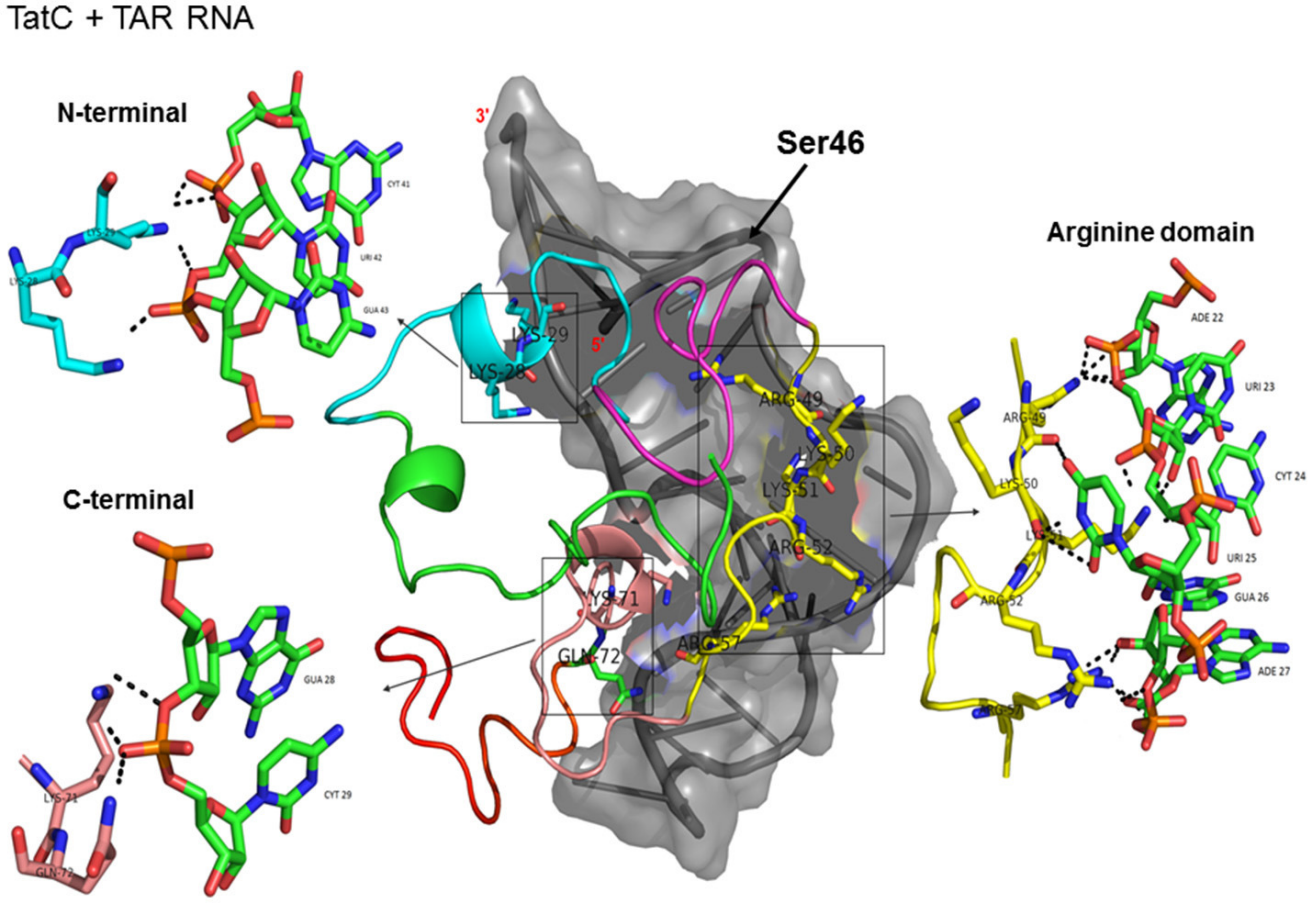
Molecular docking of wild type TatC-TAR complex. Molecular docking was carried out with the basic region of wild type TatC (coiled structure) and bulge of TAR (light blue; surface view); black-dashed lines represent H-bonds. Wild type TatC protein was represented in coiled structure [domains were represented as N-terminal region (green), cysteine rich region (blue), Core region (pink), arginine rich (yellow), glutamine rich (rose)], and TAR was represented in cartoon (blue). Docking of wild-type TatC interaction with TAR; Inset-showing N-terminal, Arginine rich region and C-terminal region interaction with TAR with H-bonds.

**Figure 3:**
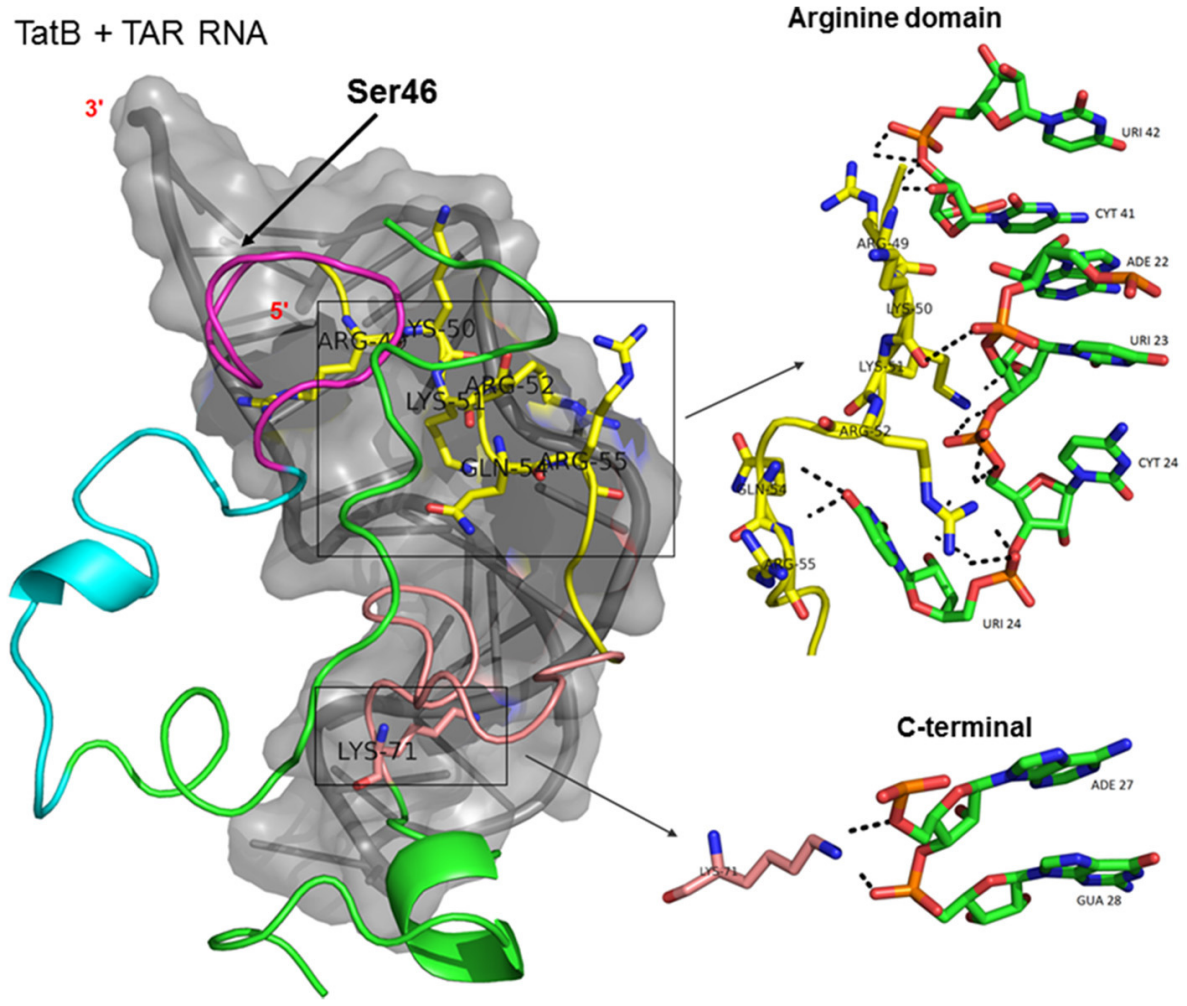
Molecular docking of wild type TatB-TAR complex. Molecular docking was carried out with the basic region of wild type TatB (coiled structure) and bulge of TAR (light blue; surface view); black-dashed lines represent H-bonds. Wild type TatB protein was represented in coiled structure [domains were represented as N-terminal region (green), cysteine rich region (blue), Core region (pink), arginine rich (yellow), glutamine rich (rose)] and TAR was represented in cartoon (blue). Docking of wild-type TatB interaction with TAR; Inset-showing N-terminal, Arginine rich region and C-terminal region interaction with TAR with H-bonds.

**Figure 4:**
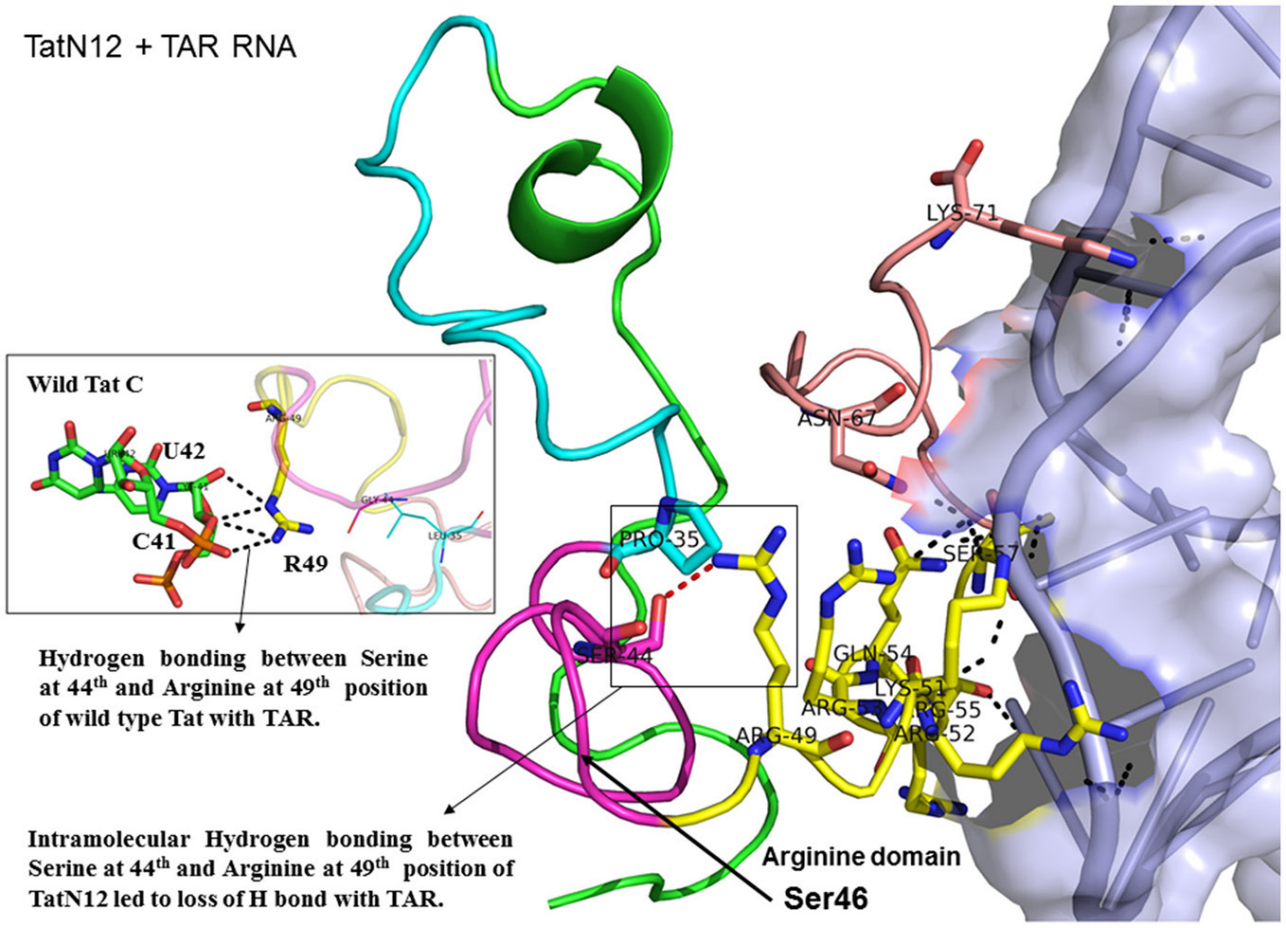
Molecular docking of TatN12-TAR complex. Molecular docking was carried out with the basic region of wild type TatN12 (coiled structure) and bulge of TAR (light blue; surface view); black-dashed lines represent H-bonds. TatN12 protein was represented in coiled structure [domains were represented as N-terminal region (green), cysteine rich region (blue), Core region (pink), arginine rich (yellow), glutamine rich (rose)] and TAR was represented in cartoon (blue). Docking of TatN12 interaction with TAR; Inset-Arg49 of wild-type Tat with U42 and C41 of TAR with H-bonds whereas TatN12 lacks H-bonds with U42 and C41 of TAR.

**Figure 5:**
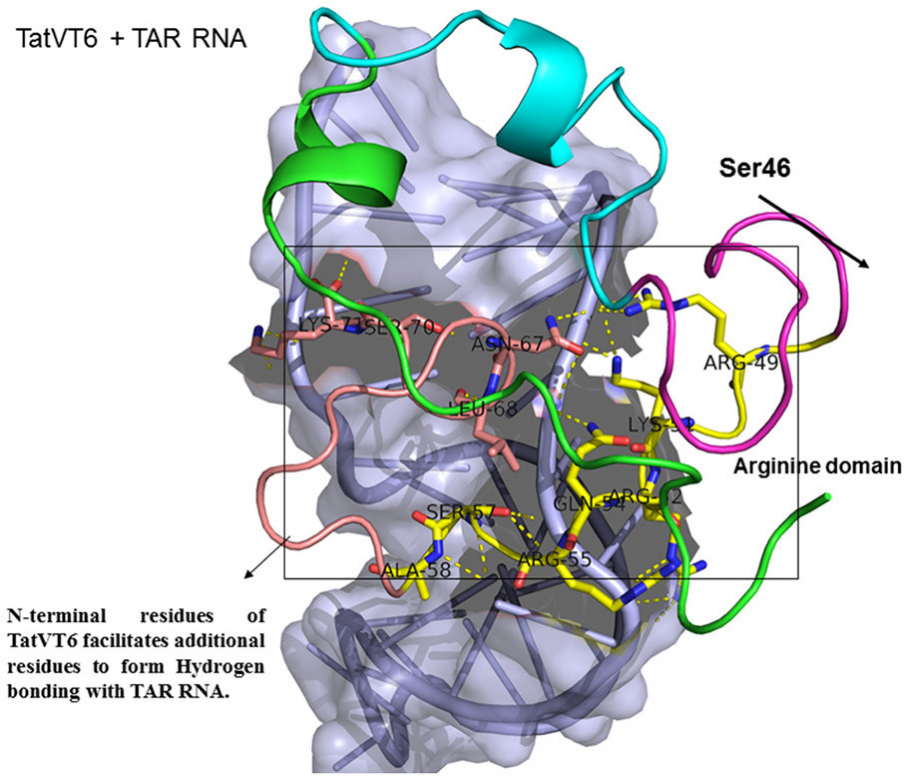
Molecular docking of TatVT6-TAR complex. Molecular docking was carried out with the basic region of wild type TatVT6 (coiled structure) and bulge of TAR (light blue; surface view); black-dashed lines represent H-bonds. TatVT6 protein was represented in coiled structure [domains were represented as N-terminal region (green), cysteine rich region (blue), Core region (pink), arginine rich (yellow), glutamine rich (rose)] and TAR was represented in cartoon (blue). Docking of TatVT6 interaction with TAR showing additional residues interactions whereas wild-type TatC lacks these residues interactions.

Docking of wild-type TatC with TAR RNA revealed that the residues in the basic region (residues 48–58) were likely to interact with the ribonucleotides of TAR. In the subtype TatC, the residues Arg49, Lys50, Lys51, Arg52, Gln54, Arg55, and Lys71 were predicted to interact with TAR (Table 1, Figure 2), whereas in the subtype TatB, the residues Lys28, Lys29, Arg49, Lys50, Lys51, Arg52, Arg57, Lys71, and Glu72 were predicted to interact with TAR (Table 1, Figure 3). Tat B resulted in a higher transactivation than Tat C which could likely be due more hydrogen bond interactions in Tat B (23 H bonds) than Tat C (16 H bonds). Two mutations Leu35Pro and Gly44Ser in TatN12 appear to change the orientation of Arg49 residue which is essential for TAR interaction and transactivation. In the TatC, Arg49 interacted with the phosphate group between ribonucleotides C41 and U42; whereas in TatN12, Arg49 residue appeared closer to Ser44 (Table 1, Figure 4); this realignment of Arg49 may be a potential reason for reduced binding affinity to TAR. TatVT6 showed relatively high binding affinity toward TAR compared to TatC. The N-terminus of TatVT6 is similar to that of subtype TatB whereas the C-terminal is similar to that of subtype TatC. The N-terminus of TatVT6 has residues namely Ser57, Ala58, Asn67, Leu68, and Ser70 that formed H interaction with TAR; the interaction of these additional residues with TAR could be the one of the reasons for enhanced TatVT6-TAR interaction *in vitro* compared to wild type TatC (Table 1, Figure 5).

**Table 1:** Hydrogen bonds formed between Tat variants and TAR from molecular docking and MD simulation experiments.

**Residue at position**	**TatC with TAR [that lacked Ser46Phe]**	**TatB with TAR [that lacked Ser46Phe]**	**TatN12 with TAR [that lacked Ser46Phe]**	**TatVT6 with TAR [B/C recombinant that lacked Ser46Phe]**	**TatD60 with TAR [with Ser46Phe]**
Lysine at 28 (K28)	–	K28 with U42 (1)	–	–	–
Lysine at 29 (K29)	–	K29 with U42 (2) + G43 (1)	–	–	Y29 with U42(2)
Histidine at 33 (H33)		–	–	–	H33 with A22(2)
Arginine at 49 (R49)	R49 with C41(3) + U42(1)	R49 with A22 (2) + U23(2) + U25(1)	–	R49 with A22(2)	R49 with A22(1)
Lysine at 50 (K50)	K50 with A22 (1)	K50 with U25(2)	–	–	–
Lysine at 51 (K51)	K51 with U23(2) + C24(1)	K51 with U25(2) + C24(2)	K51 with U23(2)	K51 with U25(2)	K51 with U23(2)
Arginine at 52 (R52)	R52 with C24(2) + U25(2)	R52 with G26(1)	R52 with U25(2)	R52 with C24(1) + U25(2)	R52 with U25(2)
Arginine at 53 (R53)	–	–	R53 with U23(1)	–	R53 with U23(2) + C24(1)
Glutamine at 54 (Q54)	Q54 with U25(1)	–	Q54 with U23(1)	Q54 with U23(1)	Q54 with C24(1) + U25(1)
Arginine at 55 (R55)	R55 with U25(1)	–	R55 with U25(1)	R55 with U25(1)	R55 with U25(2)
Serine at 57 (S57) or Arginine at 57 (R57)	–	R57 with G26(2) + A27(1) + G28(1)	S57 with C24(1)	S57 with U23(1) + C24(1) + U25(1)	S57 with U25(1)
Arginine at 58 (R58)	–	–	–	A58 with U23(1)	–
Glutamic acid at 67 (N67)	–	–	N67 with U23(1)	N67 with A22(1) + U23(1)	N67 with A22(1) + U23(1)
Leucine at 68 (L68)	–	–	–	L68 with A22(1)	–
Serine at 70 (S70)	–	–	–	S70 with G21(1)	S70 with U25(1)
Lysine at 71 (K71)	K71 with A27(1) + G28(1)	K71 with G28(1) + C29(1)	K71 with G21(2) + A22(1)	K71 with U42(3)	K71 with A27(1) + G28(3)
Glutamine at 72 (Q72)	–	Q72 with C29 (1)	–	–	–
Total number of H bonds	16	23	12	20	24

*TAR, ribonucleotides; A, adenine; U, uracil; G, guanine; C, cytosine. Numbers within the brackets denote number of H bonds formed between Tat residue and TAR ribonucleotide*.

**Figure 6:**
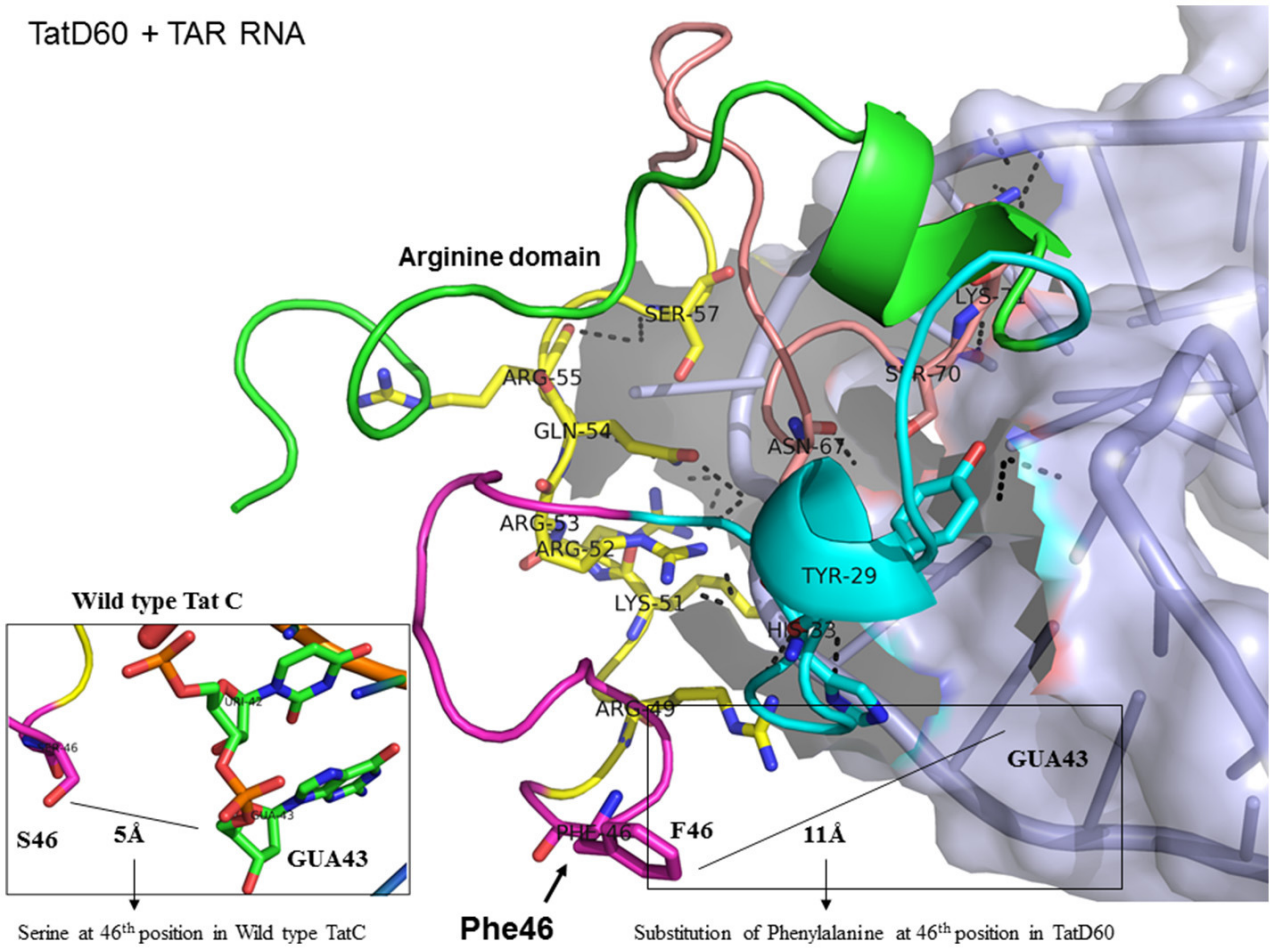
Molecular docking of TatD60-TAR complex. Molecular docking was carried out with the basic region of wild type TatD60 (coiled structure) and bulge of TAR (light blue; surface view); black-dashed lines represent H-bonds. TatD60 protein was represented in coiled structure [domains were represented as N-terminal region (green), cysteine rich region (blue), Core region (pink), arginine rich (yellow), glutamine rich (rose)] and TAR was represented in cartoon (blue). Docking of TatD60 with TAR; Inset-Ser46 of wild-type Tat with G48 of TAR at a distance of 5Å, whereas Phe46 of TatD60 with G48 of TAR at a distance of 10Å.

TatD60 (Ser46Phe) showed relatively higher binding to TAR than TatC and other Tat variants. In TatC-TAR complex, Ser46 lies about 5Å from the phosphate residue between ribonucleotides of U42 and G43, whereas the presence of a bulkier hydrophobic Phe residue at 46th position in TatD60 could likely induce conformational change in the hydrophobic core region (residues 36–47) of TatD60 protein, which in turn allow additional residue interaction at positions such as Tyr29, His33, Arg43, Ser57, and Ser70 with TAR resulting in strong H interaction between TatD60 and TAR (Table 1, Figure 6). In order to check the effect of additional mutations Glu9Lys and Ser61Arg in TatD60, docking was performed in comparison to wild type Tat C. The main interacting residues were Arg49, Lys50, Lys51, Arg52, Gln54, Arg55, Ser57, Gln60, Arg61, Ser62, and Lys71 (Table 1). Mutation Ser61Arg results in more compact binding of TAR as Arg61 is making a hydrogen bond with C19 and A20 of TAR, suggest that Ser61Arg may have also a critical role in higher binding affinity of Tat and TAR binding. This could contribute to increased binding toward TAR resulting in higher transcription *in vitro*.

### Hydrogen bond interaction between tat variants and TAR RNA

To determine the number of hydrogen bonds formed between Tat variants and TAR, we used molecular docking data. TatC-TAR was used as a reference for baseline number of hydrogen bonds formed between subtype TatC variants and TAR. TatD60-TAR had more hydrogen interaction of 24 H-bonds than TatC-TAR complex which showed 16 H-bonds. The residues Lys41, Arg49, Lys51, Arg52, Arg53, and Ser57 had similar occupancy in both TatC-TAR and TatD60-TAR complexes. In addition, residues Tyr26, Tyr29, Cys30, Ser31, Tyr47, and Ser70 also showed H-bonds in TatD60-TAR complex which could be one of the reasons for higher stability and binding affinity toward TAR leading to higher transactivation.

With respect to ribonucleotides in TAR, ribonucleotides G21, A22, U23, C24, U25, G28, G26, A27, C29, C39, U40, C41, and U42 were involved in H-bond formation with both TatC and TatD60. As expected, ribonucleotides in the bulge region of TAR binding site residues U23, C24, and U25 showed strong H-bond interaction (Roy et al., 1990). However, the residues differing in H-bond interaction between wild-type TatC and TatD60 included A22 (4 H-bonds with TatD60 while TatC did not have H-bond), U23 (5 H-bonds with TatD60, 2 H-bonds with TatC) and U25 (7 H-bonds with TatD60, 4 H-bonds with TatC) indicating strong interaction of TatD60 toward TAR. TatD60 interacted with TAR at A22 through residues Ser70, Arg5, Arg49, and Try47, whereas TatC interacted with TAR through Lys50 alone; and TatD60 at U23 through residues Lys51, Arg53, Glu67, while TatC interacted with TAR through Lys50 alone indicating strong H-interaction of TatD60 toward TAR.

## Discussion

It is known that a single mutation in the viral proteins could modulate the viral replication (Nomaguchi et al., 2014, 2016). Tat-TAR interaction is essential for LTR transactivation that account for the viral pathogenesis (Feng and Holland, 1988; Garcia et al., 1989; Puglisi et al., 1992), while any modification in the structural residues of Tat-TAR complex interaction could affect viral gene expression (Cordingley et al., 1990). Studies on Tat-TAR interaction revealed that TatN12 interaction with TAR was less or similar to that of TatC, whereas TatVT6 interacted more efficiently with TAR than other the wild-type TatC which could likely be due to differences in the structural determinants between those variants. TatD60 interacted with TAR more efficiently than other TatC which could likely be due to the presence of the Ser46Phe and Ser61Arg mutations.

Furthermore, to understand the role of Ser46Phe in TatD60 on TAR interaction, we carried out molecular docking of Tat variants with TAR. We utilized the available crystal structure (PDB ID: 5L1Z) of TAR to identify potentially important functional residues essential for Tat-TAR interaction through docking approach. It is important to determine the key residues in Tat in order to modulate Tat-TAR complex (Du et al., 2002) which will help in attenuation of viral replication (Hamy et al., 1997). Docking studies revealed the importance of unique mutations in TatN12 and TatD60, and subtype-specific variation in TatVT6 which facilitated varying number of H-bonds interacting with TAR leading to a differential binding affinity of Tat toward TAR.

This study supports previous data indicating the structural importance of lysine and arginine residues of Tat variants involved in TAR interaction (Chaloin et al., 2005). Lysine residues namely K28, K29, K50, K51, and K71 and arginine residues namely R49, R52, R53, R55, R57, R58 are highly specific target residues for hydrogen bonds with TAR. We also observed that most of lysine and arginine residues in TatD60 interacted with TAR. TatN12 showed a lower or similar level of transactivation and a weaker interaction with TAR than TatC, it appears that Gly44Ser in TatN12 led to the formation of intermolecular H-bonds between Ser44 and Arg49 that hinder the interaction of Arg49 with TAR. In TatD60, Ser46Phe produced steric hindrance that led to the exposure of cysteine-rich, core and glutamine-rich regions, allowing additional residues to interact with TAR. TatVT6 showed higher transactivation and more TAR interaction than TatC which could be due to TatB-specific variation at the transactivation region and TatC specific variation at the glutamine region which facilitated the formation of additional H-bonds to complex with TAR. The biological and clinical importance of the reported Tat mutations remains to be characterized with reference to TAR subtype C from the infected patients.

Taken together, this study illustrates the importance of structurally important key residues of Tat for modulating the specific functional activities of Tat-TAR complex. Previous reports on Tat-TAR complex indicated that hampering this complex is one of the possible targets for developing antiviral drugs (Yang, 2005; Mousseau et al., 2015) therefore, it is important to identify the important residues of Tat which would provide a novel strategy for silencing the viral gene expression. Further, this data showcases the subtype specific interaction of Tat B and Tat C with TAR for the differential transactivation abilities. Thus, this study provides valuable molecular determinants of Tat with TAR which will help in the development of targets based on Tat-TAR complex against HIV-1. Our findings elucidate the impact of mutations and subtype specific TAR interaction of Tat on viral transactivation despite current ART. Targeting these key residues to perturb Tat-TAR activity to modulate HIV-1 replication can provide novel avenues in HIV therapeutics.

## Materials and methods

### Homology modeling

Homology models of Tat protein variants were generated using the solution structure of Tat protein as a template (PDB ID: 1TAC) and a crystal structure (PDB ID: 5L1Z) using Modeller 9v8 (Eswar et al., 2007). We took the top models (High score) generated from Modeller 9v8., then the models were validated using PROCHECK (Laskowski et al., 1996) and the 3D-1D score of Verify3D (Bowie et al., 1991; Luthy et al., 1992). We ensured that no residue was present in the disallowed region (Ramachandran plot).

### Molecular docking

The molecular docking for Tat proteins and TAR RNA were carried out using HADDOCK web server (Guru Interface) (Eisenberg et al., 1997). The basic region of the Tat protein (residues 48–58) and the bulge region of the TAR (+23 to +25) were given as input active site residues to drive the docking with solvated mode activated (van Dijk and Bonvin, 2006; de Vries et al., 2007). In HADDOCK, Tat proteins (TatN12, TatVT6, and TatD60) and TAR RNA structures were first separated in space, their orientations were randomized and then the energy of the systems were minimized. The rigid-body energy minimization stage resulted in list of clusters of complexes with HADDOCK scores. The top 50 complexes with high scores were then optimized through semi-flexible simulated annealing in torsion angle space that allowed for small conformational changes of up to 2 Angstrom. In the final refinement stage, a short restrained molecular dynamic simulation in explicit solvent were conducted.

### Criteria for selection of top complex

We have used the following criteria for selecting the best complexes which included:

1. The structure had minimum energy (Low resolution, i.e., <2 Angstrom).
2. The structure have not had any mutations or missing residues.
3. The structure have not had any other ligands bound to it.
4. The structure had been densely packed conformations.
5. The structure had the secondary structure elements included.

### Molecular dynamics

TAR RNA was treated as flexible part and Tat protein was treated as semi-flexible part. From the total top 50 clusters of complexes, we have picked the top cluster (top score) for comparison analysis. We have conducted molecular dynamics for the docked structures which were found be <5 Å RMSD as described in our previous paper (Ronsard et al., 2017). The statistical significance of the docking was calculated, and the results were correlated with the precision of the models. The data showed that the protein models (<5 Å RMSD) resulted in structurally meaningful docking results, that were accurate to predict the binding residues and the hydrogen interactions of the clustered complexes of Tat-TAR.

## Ethics statement

This study design was approved by Research Project Advisory Committee, Institutional Biosafety Committee, and Institutional Ethical Committee for Human Research of University College of Medical Sciences (UCMS) and Guru Teg Bahadur (GTB) Hospital, Delhi, India, and from Post-Graduate Institute of Medical Education and Research (PGIMER), Chandigarh, India. These institutes are mentored by the National AIDS Control Organization (NACO), Ministry of Health and Family Welfare, Government of India that provides free ART to HIV-1 seropositive patients under a structured HIV/AIDS Control Program. Written informed consent was obtained from HIV-1 infected adult patients (*n* = 105) and from the guardians of HIV-1 infected children participants (*n* = 15) in this study. Blood samples were collected from HIV-1 infected patients (*n* = 120; males = 68, females = 52) registered and monitored at immunodeficiency clinics in GTB Hospital and PGIMER during the period from 2004 to 2010.

## Author contributions

LR and AB conceived and designed the experiments. LR performed the experiments. LR, TR, DR, and AB analyzed and interpreted the data. LR, TR, DR, VR, and AB contributed reagents/materials/analysis tools. LR, TR, DR, VR, and AB wrote the manuscript.

### Conflict of interest statement

The authors declare that the research was conducted in the absence of any commercial or financial relationships that could be construed as a potential conflict of interest.

## Supplementary material

Figure S1 Tertiary protein structure of Tat variants. **(A)** Homology model of Tat C; **(B)** Homology model of TatN12; **(C)** Homology model of TatVT6, and **(D)** Homology model of TatD60. Tat exon-1 containing the acidic N-terminal (green), cysteine rich (blue), core (pink), arginine rich (yellow) and glutamine rich (rose), and Tat exon-2 (red) regions represented in the coiled structure.
