# Supplementary figures and images for: In silico Analyses of Subtype Specific HIV-1 Tat-TAR RNA Interaction Reveals the Structural Determinants for Viral Activity
Source: Front Microbiol. 2017 Aug 8;8:1467. doi: 10.3389/fmicb.2017.01467 (PMC5550727; doi:10.3389/fmicb.2017.01467)

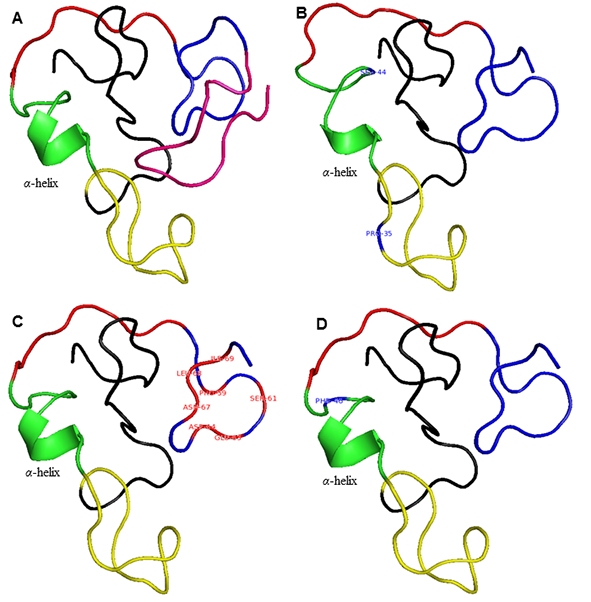

Supplement: Figure S1 — Tertiary protein structure of Tat variants. (A) Homology model of Tat C; (B) Homology model of TatN12; (C) Homology model of TatVT6, and (D) Homology model of TatD60. Tat exon-1 containing the acidic N-terminal (green), cysteine rich (blue), core (pink), arginine rich (yellow) and glutamine rich (rose), and Tat exon-2 (red) regions represented in the coiled structure. [file Image1.TIF]
